# Identification of Msp1-Induced Signaling Components in Rice Leaves by Integrated Proteomic and Phosphoproteomic Analysis

**DOI:** 10.3390/ijms20174135

**Published:** 2019-08-24

**Authors:** Ravi Gupta, Cheol Woo Min, Yu-Jin Kim, Sun Tae Kim

**Affiliations:** 1 Department of Plant Bioscience, Pusan National University, Miryang 50463, Korea; 2 Department of Botany, School of Chemical and Life Sciences, Jamia Hamdard University, New Delhi 110062, India; 3 Graduate School of Biotechnology and Crop Biotech Institute, Kyung Hee University, Yongin 17104, Korea

**Keywords:** MSP1, proteomics, phosphoproteomics, plasma membrane, plant-pathogen interaction, MAP kinase, signaling, Tandem-mass tags (TMT)

## Abstract

MSP1 is a *Magnaporthe oryzae* secreted protein that elicits defense responses in rice. However, the molecular mechanism of MSP1 action is largely elusive. Moreover, it is yet to be established whether MSP1 functions as a pathogen-associated molecular pattern (PAMP) or an effector. Here, we employed a TMT-based quantitative proteomic analysis of cytosolic as well as plasma membrane proteins to decipher the MSP1 induced signaling in rice. This approach led to the identification of 6691 proteins, of which 3049 were identified in the plasma membrane (PM), while 3642 were identified in the cytosolic fraction. A parallel phosphoproteome analysis led to the identification of 1906 phosphopeptides, while the integration of proteome and phosphoproteome data showed activation of proteins related to the proteolysis, jasmonic acid biosynthesis, redox metabolism, and MAP kinase signaling pathways in response to MSP1 treatment. Further, MSP1 induced phosphorylation of some of the key proteins including respiratory burst oxidase homologue-D (RBOHD), mitogen-activated protein kinase kinase kinase-1 (MEKK1), mitogen-activated protein kinase-3/6 (MPK3/6), calcium-dependent protein kinase (CDPK) and calmodulin (CaM) suggest activation of PAMP-triggered immunity (PTI) in response to MSP1 treatment. In essence, our results further support the functioning of MSP1 as a PAMP and provide an overview of the MSP1 induced signaling in rice leaves.

## 1. Introduction

Proteins play a central role in plant-pathogen interaction [1]. Some of the pathogen secreted proteins act as pathogen-associated molecular patterns (PAMPs) and are recognized by the plasma membrane-localized pattern-recognition receptors (PRRs) of the plants, while others function as effectors and are identified by the cytoplasmic R-gene products of the plants [2]. Recognition of PAMPs by PRR leads to the activation of pattern-triggered immunity (PTI), while recognition of effectors by the R-proteins results in the activation of effector-triggered immunity (ETI) [3]. PRRs, in general, are classified into receptor-like proteins (RLPs) and receptor-like kinases (RLKs) that contain an additional intracellular kinase domain compared to RLPs in addition to the extracellular ligand-binding domain. Chitin oligosaccharides are the well-known example of fungal PAMP which are identified by chitin elicitor binding protein (CEBiP), a RLP, and chitin elicitor receptor kinase 1 (CERK1), a RLK in rice. In addition to chitin, β-glucan, MSP1 and MoHrip1 are also emerging as fungal PAMPs, with the latter two particularly identified from *Magnaporthe oryzae* or rice blast fungus [4,5]. 

MSP1 (also referred as MoSM1) was first identified as a *M. oryzae* secreted protein in response to rice infection more than a decade ago [4]. It belongs to the cerato-platinin (CP) family which was first discovered in fungus *Ceratocystis platani* [6]. Proteins of CP family are small (150 a.a) and cysteine-rich and have been identified in a wide-range of filamentous fungi including biotrophs, hemibiotrophs, and necrotrophs [7]. The majority of the CP family proteins are secreted in the culture filtrate, however, some remains attached to the cell wall [8]. Recently, it was shown that a CP protein EPl1 from *Trichoderma harzianum* is transported through fungal cell wall and is probably involved in the interaction with host cells [9]. Moreover, other members of CP family including MSP1, FocCP1 from *Fusarium oxysporum* f. sp. *cubense* tropical race 4 (Foc TR4) and BcSpl1 from *Botrytis cinerea,* have been linked to the fungal virulence [4,8,10]. Subsequent analysis of MSP1 and FocCP1 showed that exogenous treatments of these proteins induce cell death and elicits defense responses in rice and tobacco [11,12]. Moreover, it was reported that the overexpression of MSP1 in rice confers broad-spectrum disease resistance against bacterial and fungal pathogens, including *Xanthomonas oryzae* and *M. oryzae* [13]. Moreover, phosphorylation of MAPK3/6 and oxidative burst were also observed after the MSP1 treatment in rice. As all of these responses are the hallmark of PTI, activation of these responses by MSP1 suggests its functioning as a PRR [14]. Furthermore, immunoblot analysis of MSP1 protein in rice after the *M. oryzae* infection detected MSP1 protein in rice apoplast only and not in the cytosol [12], further suggesting that MSP1 functions as a PAMP. 

Previous reports have shown that exogenous treatment of MSP1 leads to the activation of defense responses in rice; however, the details of MSP1 induced signaling and modulated pathways by MSP1 are still elusive. Previously, we used a label-free quantitative proteome analysis to understand the MSP1 induced signaling in rice and to identify its potential interacting receptors together with flg22, a well-known bacterial PAMP [14]. This study led to the identification of several proteins related to the signaling and defense; however, the receptors/receptor-like proteins (RLPs) and receptor-like kinases (RLKs) remained poorly identified because of their low abundance and difficulty in their isolation being the integral components of the plasma membrane (PM). Therefore, here we specifically enriched the PM-localized proteins together with cytosolic proteins for the enrichment of both PM-localized and intracellular receptors, followed by proteome and phosphoproteome analysis to investigate the MSP1 induced signaling in rice leaves. 

## 2. Results 

### 2.1. Quantitative Proteome Analysis

**Figure 1:**
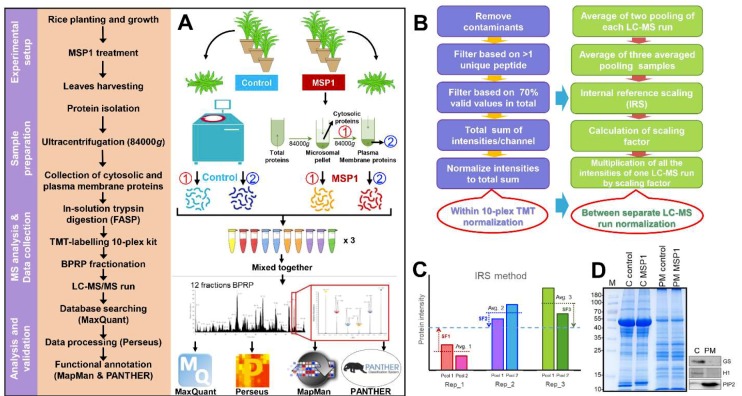
Flowchart of the methodology used for the total and plasma membrane proteome analysis. (**A**) Pipeline for the TMT based proteomics approach for proteome analysis of cytosolic and plasma membrane samples after MSP1 treatment. (**B**,**C**) Methodology for the normalization of protein intensities within 10-plex kit and between separate LC-MS runs.(**D**) SDS-PAGE and Western blotting using organelle specific marker proteins, Glutamine synthase (GS) as cytosolic marker, Histone 1 (H1) as nuclear marker and Plasma membrane intrinsic protein 2 (PIP2) as plasma membrane marker.

To understand the molecular mechanism of MSP1 induced signaling in rice leaves, protein profiles of cytosolic and PM fractions were generated using a TMT-based proteomics approach (Figure 1A–C). Total cellular proteins were isolated from the rice leaves 24 hours after MSP1 treatment and cytosolic and PM-localized proteins were fractionated using a two-phase partitioning method (Figure 1A). The purity of the extracted PM proteins was checked on SDS-PAGE and Western blots using organelle-specific marker proteins (Figure 1D). Glutamine synthase (GS), histone 1 (H1), and plasma membrane intrinsic protein 2 (PIP2) were used as cytosolic, nuclear and PM specific markers, respectively. Both GS and H1 were not detected in the PM fraction while PIP2 was highly enriched (Figure 1D), indicating high efficacy of the protocol used for the PM protein isolation and high purity of the extracted proteins. For proteome analysis, isolated proteins were subjected to in-solution trypsin digestion by the filter-aided sample preparation (FASP) method and digested peptides from 4 biological replicates and 2 identical pool samples were labeled with TMT-10 plex kit (Table 1). To increase the dynamic coverage of the PM and cytosolic proteome, TMT-labeled peptides were fractionated into 12 fractions by basic pH reverse phase chromatography using an in-house developed stage tip [15]. Altogether, 72 MS runs were performed using 12 fractions each of PM and cytosolic proteome with each fraction run in triplicate (12 × 3 = 36 for cytosolic and 36 for PM). These multiple MS runs carried out could potentially result in the introduction of MS run bias and could affect downstream analysis. Therefore, normalization of TMT data was prerequisite and was carried out at two levels, first among the biological replicates of the same sample within a 10-plex TMT kit and second among the three technical replicates represented by separate MS runs or among different 10-plex TMT kit (Figure 1B). For normalization of samples within a 10-plex TMT kit, reporter ion intensities of each protein were divided with the sum of reporter ion intensity of the respected channel. This normalization was carried out to control the differences in the starting protein amount and TMT-labeling efficiency. For the second normalization, an Internal Reference Scaling (IRS) method was followed as introduced previously [16]. For the IRS method, pooled internal references labeled with two TMT reagents were averaged and compared with the average of pooled internal references of other two runs to calculate the scaling factor. Finally, the intensities of all the proteins were adjusted with the scaling factors as depicted in Figure 1B. Pearson correlation coefficient values among the technical replicates of the same sample increased from 0.93 to 0.99 after normalization (Figure S1). 

**Figure 2:**
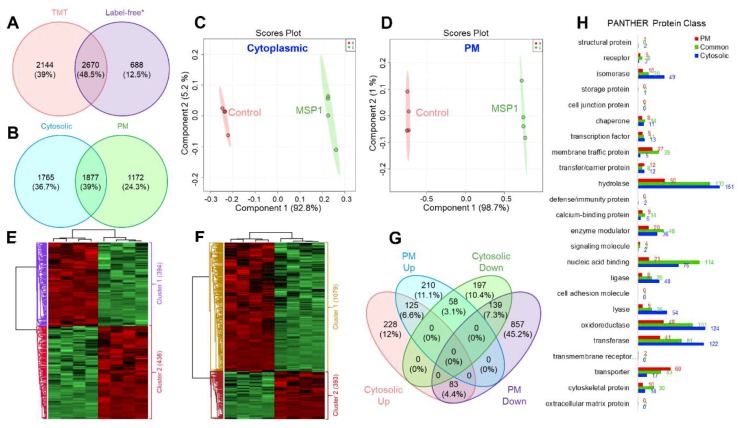
Venn diagram showing comparison of the number of identified proteins (**A**) in the current TMT based study and a previously published study; (**B**) in cytosolic and plasma membrane (PM) proteome. PLSDA scores plot showing clear separation of control and MSP1 treated proteins of (**C**) cytosolic and (**D**) PM proteome in component 1. Hierarchical clustering analysis of the differential proteins of cytosolic (**E**) and PM (**F**) fractions showing clear separation of up- and down-regulated proteins in response to MSP1 treatment. (**G**) Venn diagram showing distribution of differential proteins in the cytosolic and PM proteome after MSP1 treatment. (**H**) Functional annotation of the identified cytosolic, PM and commonly identified proteins, corresponding to Figure 2B using PANTHER protein class.

These two-step normalized intensities were then used to check the MSP1 induced changes in the cytosolic and PM proteome of rice leaves (Figure S1). In total, 6691 proteins were identified of which 3642 were from cytosolic fraction while 3049 were from PM proteome. A comparison of the number of identified proteins in the current study with our previously published study [14] utilizing a label-free quantitative proteomics approach showed 2670 (48.5%) common proteins between these two data sets with 688 (12.5%) and 2144 (39%) proteins specifically identified in the previous and current study, respectively (Figure 2A). A comparative analysis of cytosolic and PM proteome data sets showed that the 1877 (39%) of the proteins were detected both in the cytosolic and PM proteome, 1765 (36.7%) were only identified in the cytosolic fraction and 1172 (24.3%) were identified only in the PM proteome (Figure 2B). Partial least squares-discriminant analysis (PLS-DA) of control and MSP1 samples of both cytosolic and PM proteomes were separated at the component 1 accounting for 92.8% and 98.7% variations, respectively (Figure 2C,D). In addition, different biological replicates of the same sample were separated in component 2 which accounts for 5.2% of total variations in case of cytosolic proteome and 1% of total variations in case of PM proteome, indicating a high-reproducibility of the obtained data (Figure 2C,D). Student’s t-test controlled by a Benjamini–Hochberg FDR threshold of 0.05 was applied to identify the statistically significantly modulated proteins in response to MSP1 treatment. A total of 2302 differential proteins were observed of which 830 were from cytosolic fraction, while 1472 were from PM (Figure S2A,B). Of the cytosolic 830 differential proteins, 436 and 394 showed increased and decreased abundance, respectively (Figure S2C), while in case of PM 1472 differential proteins, 393 and 1079 proteins were observed to be increased and decreased after MSP1 treatment (Figure 2E,F, Figure S2D). Hierarchical clustering analysis led to the separation of differential proteins into two separate groups comprising of increased and decreased proteins (Figure 2E,F). Further cross-examination of differential proteins of cytosolic and PM fractions showed 125 and 139 common proteins that were up- and down-regulated in both the fractions, respectively. In addition, 58 proteins were observed that showed increased abundance in the PM proteome and decreased abundance in the cytosolic proteome, while other 83 proteins showed a reverse trend (Figure 2G).

### 2.2. Functional Annotation of the Identified Proteins

**Figure 3:**
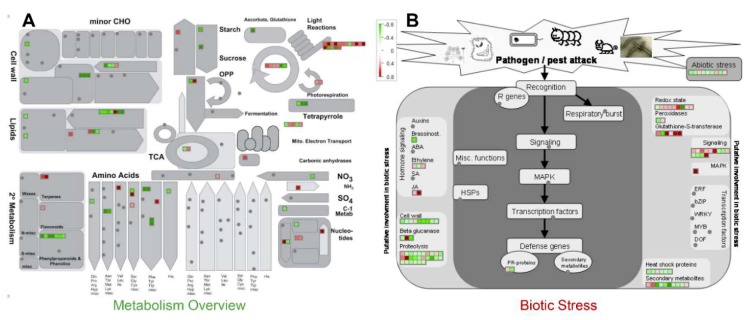
Functional annotation of the identified proteins by MapMan analysis. Differential proteins after the cutoff > 1.2 fold change for increased abundance and 0.8 fold change for decreased abundance) from both cytosolic and plasma membrane (PM) proteome analysis were used for the mapping in the metabolism overview (**A**) and biotic stress overview (**B**) categories of the MapMan. Increased and decreased expression pattern of the mapped proteins are marked by the red and green color scheme, respectively.

PANTHER protein class analysis of the identified proteins showed that the PM proteome was dominated by the transporters, while the cytosolic proteins were dominated by the hydrolases, oxidoreductases, and transferases (Figure 2H). Commonly identified proteins in both the fractions majorly included nucleic acid binding proteins and hydrolases (Figure 2H). Further, MapMan analysis showed an overall downregulation of cell wall, amino acid, tetrapyrrole, phenylpropanoids, and phenolics metabolism in response to MSP1, while an overall upregulation of light reaction, Calvin cycle nucleotide metabolism, signaling, redox regulation, and jasmonic acid signaling was observed (Figure 3A,B). Moreover, a mixed regulation of the proteins related to the proteolysis was observed. Proteins including serine carboxypeptidase-like 29, serine carboxypeptidase-like 50, NAD(P)-binding Rossmann-fold superfamily protein, and ubiquiting-conjugating enzyme 2 showed increased abundance, while others were down-regulated. In addition, an accumulation of redox associated enzymes including glutathione-S-transferases, peroxiredoxins, thioredoxins, superoxide dismutase, and catalase was observed in response to MSP1 treatment. 

Subsequently, 48 RLPs/RLKs and 7 MAP kinases (MPKs) (Tables S1 and S2) were also identified; however, except for MPK3/6, chitin elicitor binding protein (CEBiP) and chitin elicitor receptor kinase 1 (CERK1), no other protein from this list showed significant changes in response to MSP1 treatment. In order to get further functional insights and their interacting partners, interactome of these RLPs, RLKs, and MPKs was made using STRING database and the abundance pattern of each identified protein was added using Cytoscape. Interactome analysis led to the identification of MPK3/6 as a central and key player of MSP1 induced signaling in rice. MPK3/6 interacts with MKP1, CRK11, CRK10, BIR1, WRKY33, MPK6, MKS1 and MKS2. Of these, except for MKS1, MKP2, MKP1, and WRKY33, all other interacting proteins were identified in this dataset (Figure S3).

### 2.3. Phosphoproteome Analysis

As RLPs, RLKs and MPKs are regulated by phosphorylation and dephosphorylation, a phosphoproteome analysis was also carried out to investigate the MSP1 induced changes in rice leaf. Samples were harvested after 30 min and 60 min of the MSP1 treatment from three biological replicates and pooled together. Total proteins were extracted and phosphopeptides were enriched using TiO_2_ stage tips. MS analysis was carried out in three technical replicates and data were processed by MaxQuant and Perseus software. This approach led to the identification of 1906 phosphopeptides, however, to increase the reliability and accuracy, phosphopeptides that were identified in at least two of the three replicates of at least one sample were selected and used for further analysis. Using this cutoff, a total of 1214 reproducible phosphopeptides were identified, of which 1013 showed a localization probability ≥ 0.75 and score > 40 and were considered as class I phosphosites [17] (Table S3). The rest of the sites fell into class II and class III categories as per the classification given previously; yet the probability that these peptides are phosphorylated is still larger than 99% [17]. A comparison of identified phosphoproteins, cytosolic proteins and PM proteins showed 150 common proteins among all the data set (Figure S4A). In addition, 69 and 61 common proteins were identified in the phosphoproteome and cytosolic and PM proteome, respectively, with 371 proteins uniquely identified in the phosphoproteome analysis (Figure S4A). Localization prediction of the identified phosphoproteins showed that 50% of those were nuclear localized while 19% were localized to the cytoplasm (Figure S4B). A total of 13% were localized in the chloroplast and 10% were PM-localized (Figure S4B). Kinase motif analysis of the identified phosphopeptides using PhosphositePlus database showed kinase motifs for Casein kinase II (24%), 14-3-3 domain binding motif (20%), b-adrenergic receptor kinase (16%), ERK1,2 kinase substrate motif (8%) and G-protein-coupled receptor kinase 1 substrate motif (5%) (Figure S4C). 

**Figure 4:**
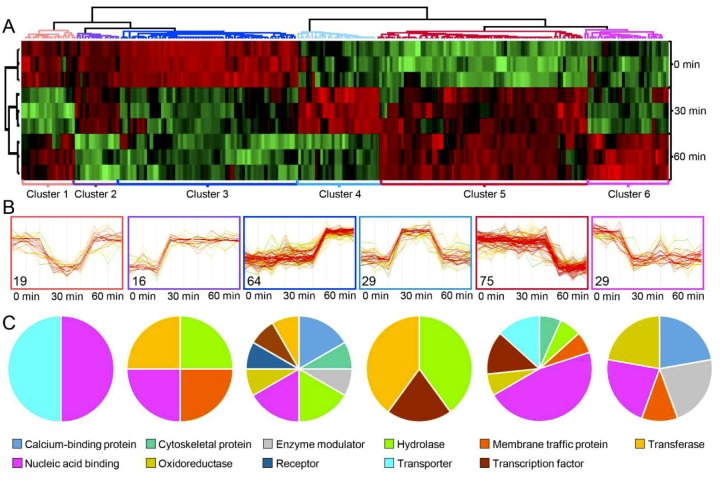
(**A**) Hierarchical clustering of the identified phosphoproteins with differential phosphosite intensities. (**B**) Expression profiles and number of identified proteins in each cluster. (**C**) Functional groups (PANTHER protein class) associated with each cluster are depicted by pie chart.

**Figure 5:**
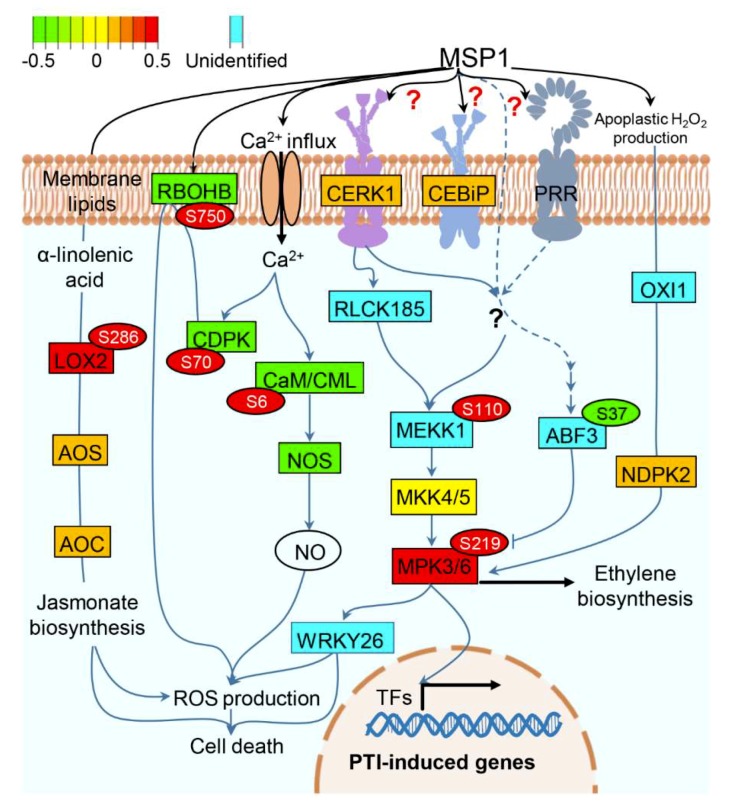
A hypothetical model showing MSP1 induced signaling in rice leaves. Abundance pattern of the identified proteins is shown by red and green color scheme while proteins not identified in this study have been highlighted by the cyan color. Phosphorylation of the respective proteins is shown in the circles with identified phosphosites. Abbreviations: CERK1: chitin elicitor receptor kinase 1, CEBiP: chitin elicitor binding protein, LOX2: lipoxygenase 2, AOS: allen oxide synthatase, AOC: allen oxide cyclase, CDPK: calcium-dependent protein kinase, CaM: calmodulin, CML: calmodulin-like, RBOHB: Respiratory burst oxidase homolog protein B, NOS: nitric oxide synthase, MEKK1: mitogen-activated protein kinase kinase kinase 1, RLCK185: receptor-like cytoplasmic kinase 185, MKK4/5: mitogen-activated protein kinase kinase 4/5, MPK3/6: mitogen-activated protein kinase 3/6, ABF3: ABA-responsive elements-binding factor 3, OXI1: oxidative signal-inducible1 (OXI1) serine/threonine protein kinase, NDPK2: nucleoside-diphosphate kinase 2, ROS: reactive oxygen species, TFs: transcription factors.

Multiple sample test, applied to the identified phosphosite intensities (Figure 4A), resulted in the identification of 232 phosphosites which changed significantly in response to MSP1 treatment (Table S3). HCL analysis of the differentially modulated phosphosites showed six clusters with clusters 1–6 containing 19, 16, 64, 29, 75, and 29 phosphosites, respectively (Figure 4B). Of these, phosphopeptides of clusters 2, 3, and 4 were of particular importance as these showed MSP1 induced phosphorylation and included many signaling and regulatory proteins (Figure 4C). Functional annotation of the identified phosphopeptides using MapMan and KEGG pathways showed increased phosphorylation of several transcription factors, RLPs, RLKs and key signaling components including MEKKK1, MAPK3/6, CDPK, among others (Figure 5, Figure S5). Moreover, phosphorylation of proteins related to protein degradation and protein modification was also increased upon MSP1 treatment (Table S3). In order to validate the phosphoproteome results, changes in phosphorylation of MAPK3/6 was also analyzed by a western blotting approach using phosphoMAPK antibodies (Erk1/2; 9101, Cell Signaling Technology). Western blots showed an increased phosphorylation of MPK3/6 from 0 min to 60 min in response to MSP1 treatment (Figure S4D), validating the results obtained from phosphoproteome analysis.

## 3. Discussion

CP family proteins have widely been identified from different fungal pathogens and include MpCP1 from Moniliophthora perniciosa [10], FocCP1 from Foc TR4 [8,11], FgCPP1 and FgCPP2 from Fusarium graminearum [18], Sp1 from Leptosphaeria maculans [19], Sm1 from Trichoderma virens [20], BcSpl1 from B. cinerea [10], Epl1 from T. harzianum [9], VdCP1 from Verticillium dahliae [21] and MSP1 from M. oryzae [13,14,15]. There is compelling evidence gathered over the years that showed involvement of these proteins in fungal virulence, with some proteins acting as elicitors, some acting as effectors, and some acting as both elicitors and effectors [6]. Emerging evidence indicates that MSP1 functions as a PAMP [12,14,22] and thus it must be recognized by the PM-localized receptors to induce the downstream signaling. Therefore, we carried out a comprehensive protein profiling of rice leaves in response to exogenous MSP1 treatment. For the in-depth proteome analysis, we performed fractionation at two steps. At first total cellular proteins were fractionated into cytosolic and PM fractions and second fractionation was carried out using TMT-labeled peptides that were divided into 12 fractions using basic pH reverse phase chromatography. This approach increased the dynamic resolution of the rice leaf samples and led to the identification of 6719 proteins which is 39% higher as compared to the number of proteins identified in the previous study [14]. The 12.5% unique proteins identified in the previous study could be related to the flag22 induced signaling as the previous approach also used a flag22 treatment in addition to the MSP1 [14]. Moreover, differences in the proteomics approach used and data analysis methods can also lead to the identification of a different number of proteins in two data sets. Further, phosphoproteome analysis led to the identification of 1214 reproducible phosphopeptides derived from 651 phosphoproteins of which 371 were uniquely identified after phosphopeptides enrichment. These uniquely identified proteins in phosphoproteome analysis could be highly low-abundant and could not be identified by total proteome analysis [23]. Functional annotation of the differential proteins showed that multiple proteins and pathways were affected by MSP1 of which some including photosynthesis (especially light reactions), cell wall modification, proteolysis, and redox regulation have been previously reported [14,22].

### 3.1. Activation of MAP Kinase Signaling by MSP1

Results obtained from both proteome and phosphoproteome analyses strongly indicate activation of MAP kinase signaling cascade in response to MSP1 treatment. Proteome analysis led to the identification of five MAP kinases including MPK1, MPK3, MPK4, MPK6, and MPK9, however, other than MPK3/MPK6, which showed increased abundance, no MPKs showed significant changes in response to MSP1. Subsequent phosphoproteome analysis showed increased phosphorylation of MEKK1/9 (MAP kinase kinase kinase1/9), MEKK-related (MAP kinase kinase kinase-related) and MPK3/MPK6 in response to MSP1 treatment. A total of three phosphosites were observed in MPK3/MPK6 at S219, T225, and Y227 of which phosphorylation of the only S219 was significantly increased in response to MSP1. Phosphorylation sites on this particular protein have not been reported to date, however, phosphosites at T225 and Y227 have been predicted in the UniProt database based on its sequence similarity with other MPKs. Moreover, no information on the phosphorylation at S219 is available and the fact that only S219 showed significantly increased phosphorylation in response to MSP1 indicate a novel regulatory mechanism of this MAP kinase regulation. Previous reports have shown that this protein functions downstream of Ca^2+^/calmodulin (CaM)-dependent protein kinase (CCaMK) OsDMI3, and participate in ABA signaling by regulating the activity of antioxidant enzymes and H_2_O_2_ production in rice [24]. In the case of MEKK1, two phosphosites were observed at S93 and S110 of which only S110 showed increased phosphorylation in response to MSP1 treatment. Phosphosites in rice MEKK1 has not been reported to date, however, in case of Arabidopsis, phosphorylation at S62 and S603 has been reported which is catalyzed by calcium/calmodulin-regulated receptor-like kinase (CRLK1) during cold stress [25]. In addition to the abiotic stresses, activation of MAP kinase signaling cascade has been reported in many plants in response to pathogen, pathogen-derived elicitors [26,27] and different phytohormones including salicylic acid [28], jasmonate [29] and, ethylene [30]. 

### 3.2. MSP1 Treatment Leads to the Activation of ROS Detoxifying Enzymes

Previous reports have shown the accumulation of reactive oxygen species (ROS) in response to exogenous treatment of CP family proteins. For example, exogenous treatment of FocCP1 and MSP1 resulted in accumulation of ROS in tobacco and rice leaves, respectively [11]. ROS burst is a critical component of PTI and thus MSP1 induced changes in the abundance of proteins involved in the redox regulation were expected. Here, we identified 51 differential proteins associated with redox regulation which were majorly increased in response to MSP1 treatment. While peroxiredoxins, thioredoxin, ascorbate peroxidase, superoxide dismutase, glutathione peroxidase, and dehydroascorbate reductase (DHAR) were increased, monodehydroascorbate reductase (MDHAR), thioredoxin and catalase were decreased in response to MSP1. A catalase was identified by the phosphoproteome analysis in which phosphosites were observed at S10 and S11; however, none of these sites had their phosphorylation significantly altered by MSP1. PAMP induced oxidative burst in Arabidopsis is mediated by the function of a PM-localized NADPH oxidase named respiratory burst oxidase homolog D (RBOHD). Its homolog in rice showed decreased abundance in response to MSP1, however, phosphoproteome results showed MSP1 induced phosphorylation of RBOHD at S32 and S750. RBOHD is phosphorylated by CDPK and surprisingly CDPK also showed a similar trend of modulation by MSP1 as RBOHD. The protein abundance of CDPK was found to be decreased upon MSP1 treatment, while its phosphorylation at S70 was increased. In addition to CDPK, flg22 dependent phosphorylation of RBOHD at S39, S343 and S347 have been shown by BIK1, a component of flagellin-sensing 2 (FLS2) immune receptor complex which is involved in the identification of flg22 [31]. Moreover, flg22 induced phosphorylation of RBOHD at S26 has also been reported [32,33]. The fact that RBOHD is phosphorylated in response to flg22 treatment, a well-known PAMP, further suggests functioning of MSP1 as a PAMP. 

### 3.3. MSP1 Induced Signaling Is Mediated by Phytohormones

Multiple proteins involved in jasmonic acid (JA), ethylene, auxin, and brassinosteroids signaling were differentially modulated by MSP1. In the case of JA, allen oxide synthase, allen oxide cyclase and three isoforms of lipoxygenase 2 (LOX2) were found to be increased by MSP1. Of the identified LOX2 isoforms, LOC_Os12g37260.1 was also found to be phosphorylated at S286 and S360 and both showed increased phosphorylation in response to MSP1. As all of these proteins participate in JA biosynthesis, increased abundance of these proteins by MSP1 suggest JA production in response to MSP1 treatment. Up-regulation of *LOX* genes and activation of JA signaling were also shown in tobacco leaves in response to FocCP1 treatment, a CP family protein from *F. oxysporum* [11]. In *Vitis rupestris*, it was shown that the jasmonates were produced only by the PAMP flg22 treatment and not by the elicitor Harpin, although the majority of the defense responses overlaps in response to these PAMP and elicitor treatments [34]. In addition to flg22, JA accumulation was also observed in potato in response to Pep-13 treatment, a PAMP from *Phytophthora* [35], further highlighting the involvement of JA in PTI responses. 

Universal stress proteins (USPs) are widely distributed in almost all the living organisms including bacteria, archaea, fungi, protozoa, plants, and mammals [36]. In plants, these proteins play a positive role in stress tolerance, especially abiotic stress with very little information on the biotic stress [36]. Here, a USP family protein was identified showing increased abundance and increased phosphorylation at S12 upon MSP1 treatment. It was shown that an OsUSP1 mediates the ethylene signaling in response to submergence stress in rice [37]. In tomato, USP is phosphorylated by the action of a Calcineurin B-like interacting protein kinase 6 (CIPK6) and regulate the CIPK6 mediated ROS generation [38]. In addition, two USP proteins from Arabidopsis (AtPHOS32 and AtPHOS34) were shown to be phosphorylated by the AtMPK3 and AtMPK6 in response to flg22 treatment in suspension culture cells [39]. It was observed that the phosphoserine was followed by a Proline and sP motif is a common substrate for MAP kinases [39]. Interestingly, we also observed the sP motif in the identified USP and thus it is highly likely that the identified USP here too is phosphorylated by the action of MPK3/6. 

In the case of abscisic acid (ABA) signaling, the majority of the identified proteins were downregulated. In addition, MSP1 induced dephosphorylation of an ABA responsive elements-binding factor 3 (ABF3) at S37 was observed, suggesting an overall negative regulation of MSP1 and ABA signaling in rice. ABF3 plays a central role in ABA signaling together with AREB1 and AREB2 and SNF1-related kinases 2 (SnRK2) mediated phosphorylation of ABF3 is crucial for the ABA signaling [40]. Recently, it was shown that the phytopathogens exploit the JA and ABA signaling pathways of plants to promote virulence. ABA induces the expression of protein phosphatases 2C (PP2Cs) through ABF/AREB transcription factors which dephosphorylate the MPK3/6. It was reported that *Pseudomonas syringae* pv. *Tomato* (*Pto*) DC3000 induces the expression of a PP2C, HAI1 in Arabidopsis that dephosphorylates the MPK3/6, thereby suppressing the MPK3/6 mediated immune responses [40]. However, Arabidopsis can overcome this HAI1 induced suppression of the MPK3/6 signaling responses by the activation of ETI [40], indicating that plants possess a mechanism to reactivate the MPK3/6 mediated signaling by blocking the ABA signaling. Similarly, dephosphorylation of ABF3 observed here, could be the result of plant defense responses to block the ABA-induced suppression of MPK3/6 mediated signaling in response to MSP1 treatment. However, confirmation of this hypothesis needs further experimentations. 

### 3.4. A Proposed Model to Elucidate the MSP1 Induced Signaling in Rice

Based on our results, we propose a model explaining the probable function of MSP1 in rice leaves (Figure 5). MSP1 may interact with CEBiP/CERK1 or to other unknown RLP(s)/RLK(s), triggering calcium influx which is sensed by CDPK and CaM/CML. Although the protein levels of CDPK and CaM/CML were decreased, these were found to be phosphorylated in response to MSP1 and it is well-known that these proteins are activated by their phosphorylation. The MSP1 induced activation of CDPK may, in turn, phosphorylates and activates the RBOHD protein which mediates the PAMP induced oxidative burst response in rice. Subsequently, binding of MSP1 to its receptor may lead to the activation of RLCK185 or any other unknown kinase functioning upstream of MEKK1, resulting in the phosphorylation of the latter. Phosphorylated MEKK1 then phosphorylates MKK4/5 which in turn phosphorylates MPK3/6, a pivotal protein which is emerging out to be a key regulator of MSP1 induced signaling in rice. Activated MPK3/6 then regulate a variety of cellular events including H_2_O_2_ production, cell death, ethylene biosynthesis, and induction of PR and other PTI-inducible genes. In parallel, MSP1 recognition by PRR(s) leads to the JA biosynthesis by elevating the abundance of LOX2, allen oxide synthase (AOS) and allen oxide cyclase (AOC) and inducing the phosphorylation of LOX2. JA thus produced may regulate various cellular processes including H_2_O_2_ production and cell death [41]. ABA is known to inhibit the SA and ethylene signaling by targeting the MPK3/6. ABA signal is transmitted through ABF3 which is activated by its phosphorylation by SnRK2. Inhibition of MPK3/6 signaling by ABA was inhibited by the MSP1 induced dephosphorylation of ABF3 by unknown phosphatase. Taken together, this model provides an overview of MSP1 induced signaling and is generated based on the data obtained here and previously published reports, however, further experimentation is required to confirm the actual functioning of this signaling pathway in response to MSP1. 

## 4. Materials and Methods

### 4.1. Plant Material, Growth Conditions, and Sample Preparation

*Oryza sativa* L. Dongjin seeds were sterilized in 0.05% Spotak solution (Bayer crop science, South Korea) overnight at 4 °C, and then washed with distilled water five times. Sterilized seeds were germinated on moist tissue paper at 28 °C in the dark and were transferred to sterilized soil in a growth chamber (70% humidity, 25 °C; a light/dark cycle of 16/8 hours) [14]. For analyzing the effect of MSP1, 4-week-old rice leaves were sprayed with 0.01% Tween-20 as control or 5 μM purified recombinant MSP1-His protein in 0.01% Tween-20. Treated leaves were harvested 30 min and 1 hour after MSP1 treatment for phosphoproteome analysis and after 24 hours for proteome analysis. 

### 4.2. Isolation of Cytosolic and PM-Localized Proteins

PM proteins were isolated from the rice leaves using a two-phase portioning method with slight modifications [42]. In brief, approximately 40 g of leaves from each sample were powdered using liquid nitrogen and homogenized in the extraction buffer containing 50 mM Tris pH 8.0, 500 mM sucrose, 10% glycerol (*w/v*), 20 mM EDTA, 20 mM EGTA, 0.6% PVP, 10 mM ascorbic acid and protease inhibitor cocktail. Homogenate was filtered through nylon cloth and centrifuged at 26,000 *g* for 25 min at 4 °C. The supernatant thus obtained was successively filtered through 63- and 34- μm filters and microsomes were pelleted down by ultracentrifugation at 84 000 *g* for 25 min at 4 °C. The supernatant thus obtained was used as the cytosolic fraction and pellet containing the microsomal proteins was dissolved in 9 mL of upper phase solution containing 5 mM potassium phosphate buffer pH 7.8, 330 mM sucrose and 2 mM DTT followed by sonication. After complete solubilization of pellet, 18 mL of lower phase solution containing 5 mM potassium phosphate buffer pH 7.8, 5 mM KCl, 300 mM sucrose, 6.4% Dextran T-500 and 6.4% PEG-3350 was added, vortexed well and incubated on ice for 5 min. phase separation was carried out by centrifugation at 2000g for 10 min. Upper phase containing PM proteins were collected and the lower phase was back extracted to maximize the yield. Finally, both the upper phases were collected, diluted five-times using deionized water and incubated on ice for 5 min. Finally, the PM proteins were precipitated 84,000 *g* for 10 min at 4 °C [42]. 

### 4.3. In-Solution Trypsin Digestion, Peptide Labeling, and Fractionation

Trypsin digestion was carried out using FASP method as described previously [43] and peptides so obtained were quantified using Pierce™ Quantitative Fluorometric Peptide Assay (Thermo Scientific, Waltham, MA, USA) following manufacturer’s protocol. For TMT-labeling, 40 μg of peptides from each sample labeled with 170 μg of TMT reagents dissolved in anhydrous CAN using a TMT 10-plex kit with each sample labeled in four replicates and two pooling samples (prepared for normalization between runs by combining 20 μg of the peptide from each individual sample). Prior to incubation of the peptides with TMT reagents, additional ACN was added to a final concentration of 30% (v/v). After incubation at room temperature for 1 hour, the reaction was quenched with hydroxylamine to a final concentration of 0.3% (*v*/*v*). Finally, all the labeled peptides from different samples and pooled references were combined together and lyophilized. Lyophilized peptides were reconstituted in 0.1% trifluoroacetic acid (TFA) containing 2% ACN and desalted using Oasis^®^ HLB 1cc (360 mg) solid-phase extraction (SPE) cartridge (Waters, Milford, MA, USA) following manufacturer’s instructions. Eluted peptides were dried down, dissolved again in 15 mM ammonium formate containing 2% ACN and fractionated into 12 fractions using an in-house developed stage tip containing C18 Empore disk membranes (3M, Bracknell, UK) and POROS™ 20 R2 reversed-phase resin (Thermo Scientific, Waltham, MA, USA), as per the method described previously [15].

### 4.4. Protein Extraction and Phosphopeptides Enrichment

For phosphoproteome analysis, control and MSP1 treated leaves (after 30 min and 1 hour of MSP1 treatment) from three biological replicates were pooled together and homogenized in the lysis buffer containing 100 mM tetraethylammonium bromide (TEAB) pH 8.5, 6 M guanidine hydrochloride and 10 mM DTT. Samples were vortexed well and incubated at 95 °C for 5 min in a dry bath. After incubation, samples were allowed to cool on ice for 15 min, sonicated for 3 min and heated again at 95 °C for 5 min. Samples were centrifuged at 12,000× *g* for 15 min at 4 °C and supernatant was used as crude protein. Protein concentration in each fraction was quantified using 2D-Quant Kit (GE Healthcare, Uppsala, Sweden). A total of 4 mg of protein from each sample were subjected to in-solution trypsin digestion by the FASP method and the peptides that were obtained were quantified by Pierce™ Quantitative Fluorometric Peptide Assay. A total of 3 mg of peptides from each sample were used for desalting and phosphopeptides enrichment using Sep-Pak^®^ plus C18 disc cartridges and High-Selecet™ TiO_2_ phosphopeptide enrichment kit (Thermo Scientific, Waltham, MA, USA), respectively, following the recommended protocol. Briefly, desalted lyophilized peptides were dissolved in the binding buffer provided with the kit, sonicated for 3 min and centrifuged to be clarified the dissolved peptides. TiO_2_ spin tips were washed with wash buffer and equilibrated with binding buffer before loading of peptides. Phosphopeptides were allowed to bind to the TiO_2_ resin followed by sequential washing with binding buffer and wash buffer. Finally, bound phosphopeptides were eluted using elution buffer and lyophilized quickly to avoid dephosphorylation of eluted phosphopeptides in the acidic elution buffer [44]. 

### 4.5. Q-Exactive MS Analysis

Lyophilized peptides were dissolved again in solvent-A (water/ACN, 98:2 *v/v;* 0.1% formic acid) and separated by reversed-phase chromatography using a UHPLC Dionex UltiMate^®^ 3000 (Thermo Scientific, Waltham, MA, USA) instrument [45]. For trapping the sample, the UHPLC was equipped with Acclaim PepMap 100 trap column (100 μm × 2 cm, nanoViper C18, 5 μm, 100 Å) and subsequently washed with 98% solvent A for 6 min at a flow rate of 6 μL/min. The sample was continuously separated on an Acclaim PepMap 100 capillary column (75 μm × 15 cm, nanoViper C18, 3 μm, 100 Å) at a flow rate of 400 nL/min. The LC analytical gradient was run at 2% to 35% solvent B (100% ACN and 0.1% formic acid) over 90 min, then 35% to 95% over 10 minutes, followed by 90% solvent B for 5 minutes, and finally 5% solvent B for 15 minutes. Liquid chromatography-tandem mass spectrometry (LC-MS/MS) was coupled with an electrospray ionization source to the quadrupole-based mass spectrometer QExactive™ Orbitrap High-Resolution Mass Spectrometer (Thermo Scientific, Waltham, MA, USA). Resulting peptides were electro-sprayed through a coated silica emitted tip (Scientific Instrument Services, Ringoes, NJ, USA) at an ion spray voltage of 2000 eV. The MS spectra were acquired at a resolution of 70,000 (200 *m*/*z*) in a mass range of 350-1650 *m*/*z*. The automatic gain control (AGC) target value was 3 × 10^6^ and the isolation window for MS/MS was 1.2 *m*/*z*. Eluted samples were used for MS/MS events (resolution of 35,000), measured in a data-dependent mode for the 15 most abundant peaks (Top15 method), in the high mass accuracy Orbitrap after ion activation/dissociation with Higher Energy C-trap Dissociation (HCD) at 32 collision energy in a 100-1650 *m*/*z* mass range. The maximum ion injection time for the survey scan and MS/MS scan was 30 ms and 120 ms, respectively [46]. The mass spectrometry proteomics data have been deposited to the ProteomeXchange Consortium via the PRIDE [47] partner repository with the dataset identifier PXD014758.

### 4.6. LC-MS/MS Data Analysis for Proteomic Comparisons

The acquired MS data were analyzed with MaxQuant (ver. 1.5.3.30) [48]. MS/MS spectra were searched with the integrated Andromeda search engine against the rice protein database (88, 647 entries) and 248 common contaminant proteins. Trypsin specificity was required and a maximum of two-missed cleavages was allowed [49]. Carbamidomethylation of cysteine residues was set as fixed modification while TMT-labeled N-term, oxidation of methionine and protein N-terminal acetylation as variable modifications in case of TMT-based proteome analysis and TMT-labeled N-term was replaced with the phosphorylation of Ser, Thr, Tyr residue (phosphoSTY) in case of phosphoproteome analysis [50]. A minimum peptide length of six amino acids was specified and “match between runs” (MBR) was enabled with a matching time window of 0.7 min. The allowed mass deviation was 4.5 ppm for peptides and 20 ppm for fragments [14]. Peptide-spectrum-matches and proteins were retained if they were below a false discovery rate of 1%. Statistical analyses, hierarchical clustering analysis (HCL), and principal component analysis (PCA) were carried out using Perseus software (ver. 1.5.8.5) [51]. Hits were only retained if they were quantified in at least 70% of the total replicates. For phosphoproteome analysis, phosphopeptides that were reproducibly identified in at least two out of three replicates of at least one sample with score >40 and delta score >7 were considered as valid identification and used for the further analysis. Missing values imputation of protein intensities were performed from a normal distribution (width: 0.3, down shift: 1.8). Multiple sample test (ANOVA) threshold of 0.05 was applied to identify the significant differences (≥1.5 fold change) in the protein abundance [51].

### 4.7. Bioinformatics Analysis

The MapMan program, version 3.6.0 RC1, was used for pathway analysis [52]. Proteins fold change values were transformed into Log_2_ fold change, and their means were calculated. These non-redundant proteins or genes were classified into MapMan BINs and their annotated functions were visualized using the MapMan program by searching against *Oryzae sativa* Osa_MSU_v7 mapping. Principal component analysis (PCA) was performed using MetaboAnalyst [53]. Interactome analysis was performed using Cytoscape combined with a STRING application [54,55].

## 5. Conclusions

Plant-pathogen interaction is mediated by the secretion of various proteins from both the partners which interact with each other and determine the fate of their relationship. Some of the pathogen secreted proteins function as PAMPs, while others act as effectors and function by compromising the host immunity. MSP1 is one recently identified *M. oryzae* secreted protein that elicits the defense responses in rice. To gain an insight into the molecular action of MSP1 induced signaling in rice, here, we employed a systems biology approach to understanding the molecular mechanism of MSP1 induced signaling in rice. Proteome and phosphoproteome results obtained here shed a light on the signaling components affected or activated by MSP1 treatment. In essence, our results further emphasize that MSP1 functions as a PAMP and evidence in support of the same included (1) MSP1 induced signaling is mediated by phosphorylation of MPK3/6 and activation of MAP-kinase signaling pathway, (2) MSP1 induced activation of JA biosynthesis enzymes which probably results in the JA production, (3) MSP1 induces phosphorylation of RBOHB, a key enzyme mediating PAMP induced ROS-burst in rice. 

## Figures and Tables

**Table 1:** Summary of the sample labeling and identified proteins in the cytosolic and plasma membrane (PM) proteome fraction in control and MSP1 treated rice leaves.

TMT plex & technical replicates		Sample Labeling		Number of identified proteins		Total number of identified peptides		Average Sequence Coverage/protein	
		TMT Reagent	Sample Info	Cytosolic	PM	Cytosolic	PM	Cytosolic	PM
TMT plex set 1	Technical Replicate 1	126	Control_1	3329	3405	23,680	22,287	21.06	19.24
		127N	MSP1_1	3342	3397				
		127C	Control_2	3349	3416				
		128N	MSP1_2	3349	3410				
		128C	Control_3	3340	3418				
		129N	MSP1_3	3336	3419				
		129C	Control_4	3338	3424				
		130N	MSP1_4	3339	3416				
		130C	Pooling 1	3344	3421				
		131	Pooling 2	3340	3413				
TMT plex set 2	Technical Replicate 2	126	Control_1	3257	3432	23,672	22,324	21.1	19.32
		127N	MSP1_1	3281	3433				
		127C	Control_2	3273	3448				
		128N	MSP1_2	3271	3430				
		128C	Control_3	3274	3458				
		129N	MSP1_3	3286	3445				
		129C	Control_4	3271	3450				
		130N	MSP1_4	3275	3439				
		130C	Pooling 1	3289	3449				
		131	Pooling 2	3270	3442				
TMT plex set 3	Technical Replicate 3	126	Control_1	3326	3423	23,841	22,358	21.28	19.34
		127N	MSP1_1	3348	3420				
		127C	Control_2	3331	3426				
		128N	MSP1_2	3344	3419				
		128C	Control_3	3332	3433				
		129N	MSP1_3	3335	3429				
		129C	Control_4	3321	3444				
		130N	MSP1_4	3335	3429				
		130C	Pooling 1	3340	3421				
		131	Pooling 2	3338	3431				

## Supplementary Materials

Supplementary materials can be found at https://www.mdpi.com/1422-0067/20/17/4135/s1.
